# Dynamic Alterations of Functional Systems in Alzheimer's Disease: A Co‐Activation Pattern Analysis

**DOI:** 10.1002/hbm.70509

**Published:** 2026-03-26

**Authors:** Pan Wang, Mengfan Xue, Yingyin Mao, Chunyan Wang, Xing Yao, Bharat B. Biswal

**Affiliations:** ^1^ The Clinical Hospital of Chengdu Brain Science Institute, MOE Key Laboratory for Neuroinformation, Center for Information in Medicine, School of Life Science and Technology University of Electronic Science and Technology of China Chengdu China; ^2^ Department of Biomedical Engineering New Jersey Institute of Technology Newark New Jersey USA

**Keywords:** Alzheimer's disease, co‐activation pattern, functional networks, graph theory analysis, mild cognitive impairment

## Abstract

While resting‐state brain dysfunctions have been extensively investigated in Alzheimer's disease (AD), the dynamic alterations of functional systems remain poorly understood. We employed co‐activation pattern (CAP) analysis to characterize the functional‐state alterations in 243 participants using resting‐state fMRI data and applied graph theory analysis to estimate corresponding topological properties. The CAP analysis identified five distinct brain states across groups: State 1 (limbic network dominated), State 2 (dorsal attention network (DAN) and central executive network dominated), State 3 (default mode network and central executive network dominated), State 4 (somatomotor network and ventral attention network dominated), and State 5 (DAN, sensorimotor, and visual networks dominated). Compared to cognitively unimpaired individuals, State 3 demonstrated significantly reduced persistence and resilience in both mild cognitive impairment (MCI) and AD groups. Additionally, both clinical groups (MCI and AD) exhibited decreased transitions from State 2 to State 5 and reduced self‐transitions within State 3. Graph theory analysis revealed that compared to cognitively unimpaired individuals, MCI and AD individuals had increased node degree centrality and node efficiency, alongside decreased node local efficiency in regions within the default mode network (DAN) and visual network, which corresponded well with CAP analysis results. Our findings provide a multiscale framework linking dynamic state instability to static network reorganization, advancing understanding of the dynamic functional alterations underlying cognitive decline in AD spectrum disorders.

## Graphical abstract

- State 3 exhibited significantly reduced persistence and resilience in both MCI and AD compared to CU.
- Individuals with MCI and AD showed reduced transition probability from State 2 to State 5 and decreased self‐transitions in State 3.
- Graph theoretical results corresponded well to the findings of the above CAP analysis.

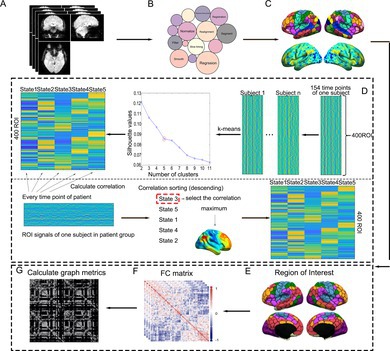

## 1 Introduction

Alzheimer's disease (AD) is an incurable neurological illness affecting an estimated 5% of men and 6% of women over the age of 60 worldwide (World Health Organization 2011). The disorder is characterized by a progressive deterioration not only of cognitive function, including memory, language, attention, and speed of perception, but also of functional ability (Backman et al. 2004; Liddell et al. 2007; Sanabria‐Castro et al. 2017), thereby impairing the patients' and caregivers' quality of life (Green et al. 2019; López‐Ortiz et al. 2021). Mild cognitive impairment (MCI), a transitional state between normal aging and dementia, is widely regarded as a prodromal stage of AD. Clinically, MCI is characterized by mild impairments in memory or other cognitive domains that do not significantly interfere with activities of daily living (Petersen et al. 2014). Previous studies have indicated that individuals with MCI progressed to AD at an annual rate of approximately 10%–15% (Allison et al. 2014), whereas the probability of AD diagnosis in cognitively unimpaired (CU) older adults remained only 1%–2% (Bischkopf et al. 2002). Consequently, the identification of MCI and AD is critical for delaying disease progression, and functional magnetic resonance imaging (fMRI) provides unique advantages in elucidating brain functional alterations associated with these conditions.

Owing to the unique advantages of fMRI in exploring brain functional alterations, recently increasing studies have utilized fMRI data to investigate the neuroimaging abnormalities of AD. Kang et al. (2024) demonstrated varying degrees of atrophy in the hippocampus, frontal cortex, and occipital regions in both individuals with MCI and AD. Furthermore, individuals with MCI and AD exhibit significantly weakened functional connectivity within key brain networks such as the default mode network (DMN), particularly in regions including the posterior cingulate cortex and prefrontal cortex (Wang et al. 2024; Buckner et al. 2008). This reduced functional connectivity is closely associated with memory deficits and declines in other cognitive functions. Studies have demonstrated graded reductions in functional connectivity across DMN, central executive network (CEN), and salience network in individuals with MCI and AD (Bublatzky et al. 2020). Moreover, global assortativity is significantly lower in individuals with MCI and AD compared to CU, suggesting a shift toward more fragmented and vulnerable network architectures (Li et al. 2024, 2025).

To capture the spatiotemporal patterns in resting‐state fMRI data and comprehensively characterize the dynamic changes of brain functional activity, the co‐activation pattern (CAP) method has been proposed to identify the transient co‐activation modes across brain regions/networks by examining single time frames (An et al. 2024). Unlike conventional sliding‐window approaches, CAP analysis can capture the dynamic functional activity at finer temporal resolutions by extracting meaningful information from individualized time points (Zheng et al. 2023). In schizophrenia research, CAP has revealed aberrant dynamic transitions in patients' brain states, which significantly correlate with symptom severity (Peng et al. 2023). Furthermore, CAP analysis in major depressive disorder has identified distinct state transition patterns compared to healthy controls, particularly involving core networks such as the frontoparietal network (Liu et al. 2023). However, dynamic brain network alterations during the clinical progression of AD remain incompletely elucidated.

We hypothesize that individuals with MCI and AD will exhibit pronounced deficits in the dynamic network disruptions, with MCI exhibiting less severe disruptions compared to AD. To this aim, we leverage the resting‐state fMRI data of AD, MCI, and CU cohorts to characterize stage‐specific alterations in brain state dynamics. Employing the data‐driven k‐means clustering method, we quantify recurrent CAPs and their temporal metrics (e.g., fraction of time and transition frequency) to investigate the dynamic functional alterations in individuals with MCI and AD.

## 2 Methods

### 2.1 Neuroimaging Dataset

All neuroimaging data in this study were obtained from the OASIS‐3 dataset (https://www.oasis‐brains.org/), collected across several ongoing studies in the Washington University Knight Alzheimer's Disease Research Center. All the neuroimages collected from OASIS‐3 datasets were original and did not undergo fMRI preprocessing and analysis. Additionally, all 243 participants signed the informed consent before the experiment. The disease stage was evaluated according to the clinical dementia rating (CDR) score. The 243 participants consisted of 53 individuals with AD (CDR = 1; Males/females: 33/20; Age: 75.89 ± 8.56 years), 90 individuals with MCI (CDR = 0.5; males/females: 48/42; age: 74.76 ± 7.67 years), and 100 CU matched for age, gender and education (CDR = 0; males/females: 50/50; Age: 74.41 ± 8.23) (LaMontagne et al. 2019). The detailed demographic and clinical information are described in Table 1.

**TABLE 1:** Demographics and clinical characteristics of participants.

	CU	MCI	AD	*p*
Number	100	90	53	
Age (years)	74.41 ± 8.227	74.76 ± 7.668	75.89 ± 8.555	0.494^a^
Gender (M/F)	50/50	48/42	33/20	0.347^b^
Education (years)	14.32 ± 1.76	14.59 ± 3.02	14.75 ± 3.20	0.844^a^
Handedness (L/R)	0/100	0/90	0/53	
MMSE	28.86 ± 1.318	25.99 ± 2.882	22.08 ± 4.057	< 0.001^a^

Abbreviations: F, female; L, left; M, male; MMSE, mini‐mental state examination; R, right.

^a^ Kruskal–Wallis test.

^b^ Chi‐square test.

### 2.2 The fMRI Data Acquisition

Neuroimages were acquired with Siemens TIM Trio 3 T MR scanners, and fMRI data consisted of 164 volumes with the following parameters: repetition time = 2200 ms, echo time = 27 ms, flip angle = 90°, number of slices = 33, slice thickness = 4 mm, and voxel size = 4 × 4 × 4 mm^3^. Anatomical images consisted of 176 slices, and repetition time = 2400 ms, echo time = 3.16 ms, flip angle = 8°, voxel size = 1 × 1 × 1 mm^3^, and slice thickness = 1 mm. More detailed scanning parameters were described elsewhere (LaMontagne et al. 2019).

### 2.3 Preprocessing Steps of Functional Images

**FIGURE 1:**
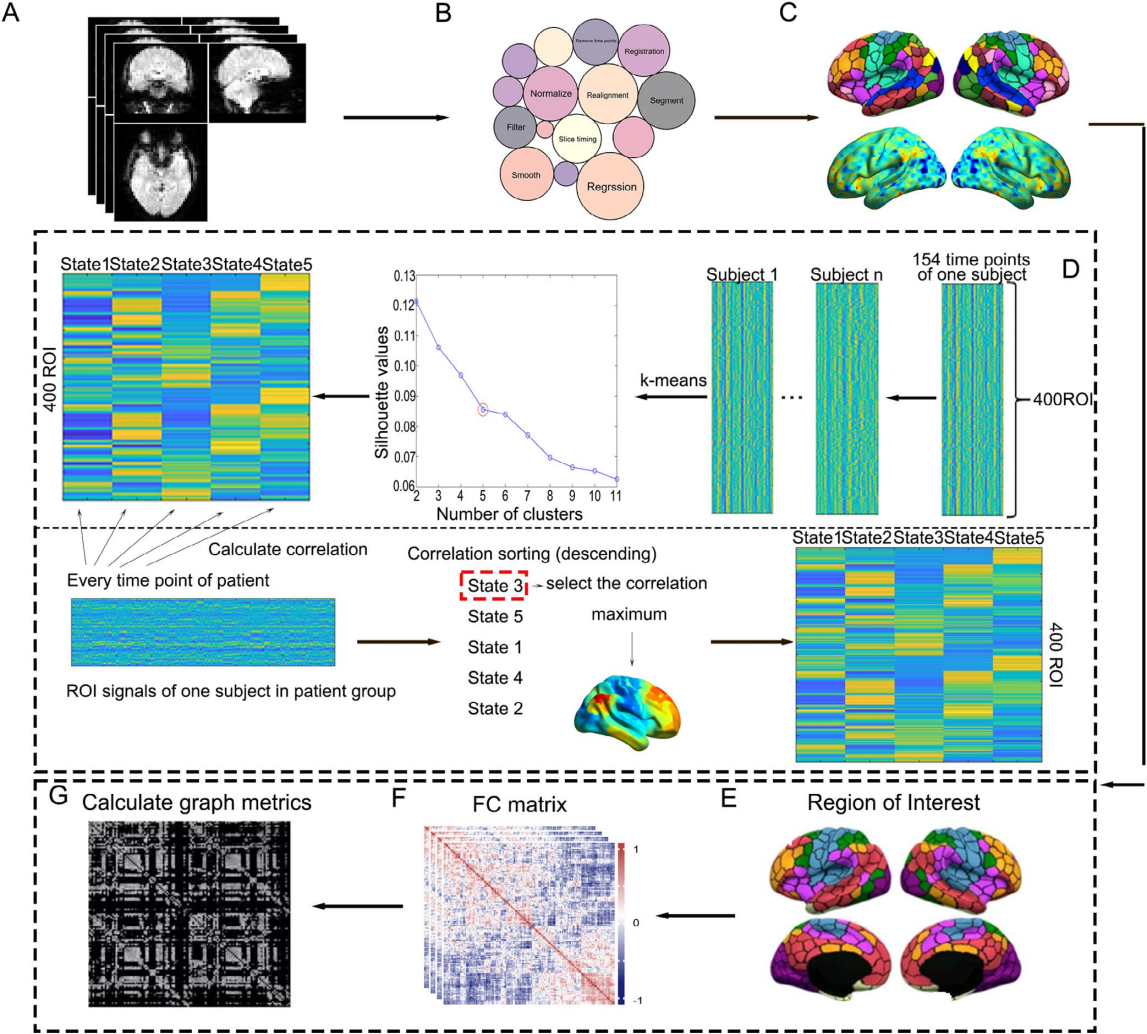
An illustration of the entire analysis process. (A) rs‐fMRI data. (B) Neuroimaging preprocessing steps. (C) The mask used to extract time series in CAP (upper part) and the operation to extract time sequences (lower part). (D) The process of CAP. BOLD signals were extracted from 400 cortical ROIs. *K*‐means clustering was applied to identify dynamic CAP. Group‐level CAP for other cohorts was derived by correlating individualized time points with the CU group's cluster centroids, ensuring cross‐group comparability of spatiotemporal network configurations. Spatiotemporal features were analyzed to capture dynamic brain activity differences. (E) Region of interest in graph theoretical analysis. (F) Calculating FC matrix. (G) Graph metrics analysis.

All neuroimaging datasets that we download are original and have not undergone preprocessing. The fMRI preprocessing was conducted using the DPARSF (Data Processing Assistant for Resting‐State fMRI, http://rfmri.org/DPARSF) and SPM (http://www.fil.ion.ucl.ac.uk/spm/software/spm12) packages. The preprocessing pipeline, illustrated in Figure 1B, included the following steps. The first 10 volumes were discarded for each participant to ensure magnetic field stabilization. Slice timing correction was applied to temporally align all slices within individual volumes. Head motion correction was performed through a six‐parameter rigid body realignment to the mean functional image. High‐resolution T1‐weighted anatomical images were co‐registered to the mean functional image. Functional images were normalized and resampled to 3‐mm isotropic voxels in Montreal Neurological Institute space. A brain mask was applied to exclude non‐brain tissue. Nuisance signal regression incorporating 24 head motion parameters, mean white matter, and cerebrospinal fluid signals was then performed. Then, we removed the linear trends and performed temporal filtering using a bandpass filter of 0.01–0.1 Hz. The functional images were smoothed using an 8‐mm full‐width at half‐maximum Gaussian kernel to enhance the signal‐to‐noise ratio and accommodate inter‐subject anatomical variability.

### 2.4 Co‐Activation Patterns Analysis

After the neuroimaging preprocessing analysis, the blood oxygen level dependent (BOLD) signals were extracted using the regions of interest (ROIs) consisting of 400 cortical regions from Yeo's 7‐network parcellation (Schaefer et al. 2018) (Figure 1C). For each participant, we performed the *Z*‐scored analysis to standardize the signal intensities for each ROI. Specifically, we first calculated the mean and standard deviation for each time series, and then subtracted the mean and divided by the standard deviation to obtain the standardized time series. The *k*‐means clustering algorithm was applied to the *Z*‐scored three‐dimensional matrix (100 CU participants × 154 time points × 400 ROIs) with “correlation” parameter instead of the usual “sqeuclidean,” which partitioned the matrix into *k* clusters based on the similarity of BOLD signal patterns across different brain regions. The similarity between distinct BOLD signals was quantified using the Pearson correlation coefficient, which assesses the linear relationship between two variables. The silhouette score was calculated to evaluate the clustering results for different *K* values, and then the optimal number of clusters was determined by using the elbow criterion (Figure S1). After performing *k*‐means clustering analysis, we identified the time series of five clusters. For each cluster, we extracted the corresponding time series and calculated their averaged values, generating a vector (400 [ROIs] × 1). Subsequently, we divided each element by the standard deviation of all elements in the vector, resulting in the normalized CAP maps.

The primary objective of this study is to quantify how disease changes the dynamic functional architecture in the brain, suggesting that a stable reference benchmark is necessary to represent the normal functional range. The CU‐driven CAPs analysis enables us to directly calculate the patient's brain deviations in dynamic metrics compared to CU. Therefore, the clustering analysis for the patient group was performed using a correlation‐based approach, incorporating the CAP results from the CU group as a reference. Specifically, BOLD signals in the patient group were correlated with the CAP results from the CU group. For each time point, similarity results were sorted in descending order, and the most similar CAP in CU was assigned as the cluster label for that time point. This approach allows for identifying CAP in the patient group that is similar to or different from those in CU (Figure 1D), resulting in which distinct CAP capture the dynamic fluctuations in brain activity over time. These patterns were then characterized by spatial distribution and temporal dynamics, providing insights into the brain's functional organization and its responses to various cognitive states.

### 2.5 Temporal Dynamic Metrics

To evaluate the dynamical properties within/between CAP states, five indicators are calculated. We set the sequence of states as *S* = [*s*_1_, *s*_2_, …, *s*_*T*_], where *s*_*t*_ denotes the state label at time *t*, and *T* is the total number of time points. Counts refer to the total number of times that each CAP state is detected throughout the entire scan. It measures the frequency of a specific pattern in brain activity occurring over the whole time series. For state *k*,

$$\text{Counts}\;\left(K\right)=\sum_{t=1}^{T}\left(s_{t}=k\right)$$

The fraction of time refers to the total time ratio occupied by a specific CAP state throughout the entire scanning time series. It reflects the overall temporal resources allocated by the brain to a particular pattern. For state *k*,

$$\text{Fraction of time}\;\left(k\right)=\frac{\text{Counts}\left(k\right)}{T}$$

Dwell time refers to the average time (in TRs) that the brain maintains a specific CAP state after entering it. It measures the stability or viscosity of the state. For state *k*,

$$\text{Dwell time}\;\left(k\right)=\frac{1}{N_{k}}\sum_{i=1}^{N_{k}}L_{k,i}$$

where $N_{k}$ is the number of consecutive segments belonging to state *k* in the state sequence *S*. $L_{k,i}$ is the length of the *i*th consecutive segment in the state *k*.

Resilience refers to the conditional probability that the brain remains in state *k* at the next time point *t* + 1, given that it is in state *k* at any given time point *t*. It directly quantifies the self‐sustaining tendency of states. For state *k*,

$$\text{Resilience}\;\left(k\right)=\frac{n\left(s_{t}=k⋂s_{t+1}=k\right)}{n\left(s_{t}=k\right)}$$

where *n* is the number of time points that meet the condition.

The transition probability matrix is a *K* × *K* matrix, where the element ${\text{Transition probability}}_{\mathrm{ij}}$ represents the probability of transitioning to state *j* in the next time step, given the current state is state *i*. It is an index to evaluate the dynamic flexibility, state switching path, and overall control strategy of the brain. The formula is as follows:

$$\text{Transition probability}\;\left(i,j\right)=\frac{C_{\mathrm{ij}}}{\sum_{j=1}^{K}C_{\mathrm{ij}}}$$

where $C_{\mathrm{ij}}$ is the number of transitions from state *i* to state *j*, and *K* is the total number of states.

### 2.6 Graph Theoretical Analysis

To investigate the abnormal network structure corresponding to the altered CAP parameters, graph analysis was performed using the Gretna software (https://helab.bnu.edu.cn/gretna/). The whole‐brain time series were extracted based on Yeo's atlas of 400 ROIs. Functional connectivity matrices were computed from the extracted time series to facilitate subsequent analyses. To ensure that all edges in the graph were significantly different from zero, we first calculated the correlation coefficient at the significant threshold of *p* = 0.05. Subsequently, edges with strengths below the statistical significance threshold were removed. The number of edges remained divided by the total number of edges (i.e., *n* × (*n* – 1)/2, where *n* denotes the number of nodes) defined the sparsity of the graph (Li et al. 2020). For each element of the matrices, we calculated the absolute values of all negative correlations in the graph theoretical analysis.

We employed a two‐step approach to establish the minimum value for the connectivity matrix. First, the mean degree over all nodes of the binarized network was larger than 2 × log(*n*) (Bullmore and Bassett 2011). We identified the minimum threshold value that ensured the presence of a giant cluster in the network. This step was achieved by iteratively decreasing the threshold from one and calculating the size of the largest connected component (giant cluster) until it encompassed all nodes. Second, the number of nodes in the giant connected cluster (i.e., the largest subgraph) was greater than 70% × *N* (Li et al. 2020). The corresponding threshold value was recorded as the minimum threshold. Accordingly, the range of sparsity threshold was 0.07–0.54 for the whole brain, with a common step of 0.01.

Several nodal parameters were calculated to characterize the network's topological properties, including the degree centrality, node efficiency, and node‐local efficiency. Degree centrality measures the number of direct connections in a brain region. High‐degree nodes act as network hubs, facilitating global information integration. Degree centrality alterations can signal brain disorders (Bullmore and Sporns 2009). The formula is as follows:

$$\text{Degree centrality}=\frac{1}{N}\sum_{i=1}^{N}D_{i}$$

where *N* is the total number of nodes in the network, and *D*_*i*_ is the degree of node *i*.

Node efficiency assesses a node's ability to communicate with others via shortest paths. The area under the curve of node efficiency reflects overall information‐transfer flexibility. Higher node efficiency links to better cognitive performance (Latora and Marchiori 2001). The formula is as follows:

$$\text{Node efficiency}=\frac{1}{N}\sum_{i\neq j}\frac{1}{d_{\mathrm{ij}}}$$

where *N* is the total number of nodes in the network, and $d_{\mathrm{ij}}$ is the shortest path length from node *i* to node *j*.

Node local efficiency evaluates information processing within a node's local subnetwork. Brain regions with high node local efficiency are crucial for specific local tasks (Sporns et al. 2004). The formula is as follows:

$$\text{Node local efficiency}=\frac{1}{N}\sum_{i=1}^{N}E_{\left(\text{local}\right)}\left(i\right)$$

$$E_{\left(\text{local}\right)}\left(i\right)=\frac{1}{k_{i}\left(k_{i}-1\right)}\sum_{j,k\in N_{i}}\frac{1}{d_{\mathrm{jk}}^{N_{i}}}$$

where $N_{i}$ is the set of neighbors of node *i*, $k_{i}$ is the degree of node *i*, and $d_{\mathrm{jk}}^{N_{i}}$ is the shortest path length between node *j* and *k* in the neighbor subgraph.

### 2.7 Statistical Analysis

Nonparametric tests were selected for their robustness against non‐normality and heterogeneity of variances, providing a detailed comparison of between‐group differences. The study performed the statistical analysis using the Kruskal–Wallis test and the Mann–Whitney *U*‐test. Specifically, the Kruskal–Wallis test was used to examine the three‐group differences in CAP analysis and graph theoretical analysis, controlling for the effect of age, gender, and education. The obtained *p* values were then corrected using the false discovery rate (FDR) method with *p* < 0.05. A post hoc Mann–Whitney *U*‐test was performed to examine the pairwise differences between CU, MCI, and AD groups.

## 3 Results

### 3.1 CAPs and Brain States

**FIGURE 2:**
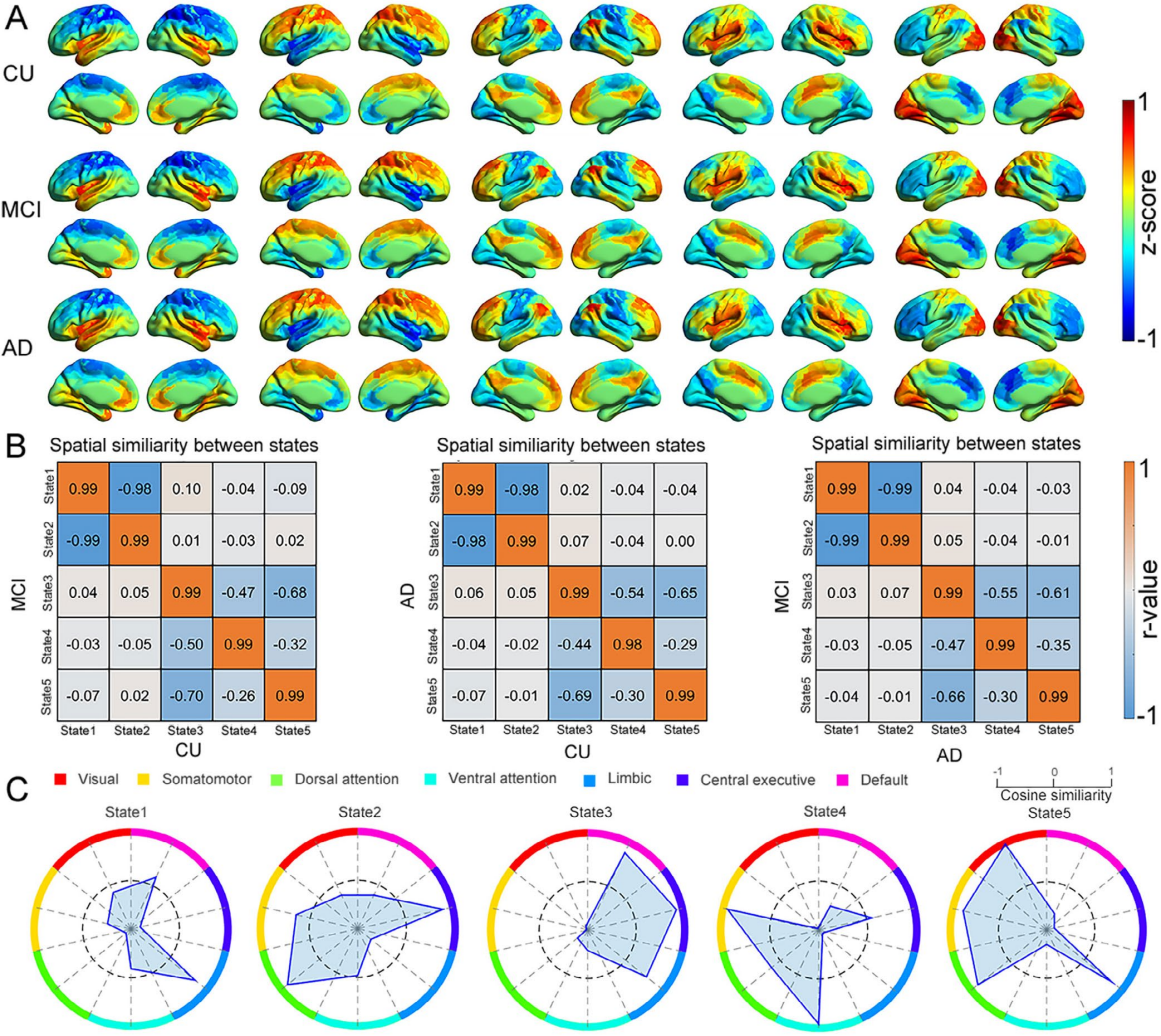
Five distinct brain states in three groups. (A) The five identified CAP for each group. (B) Spatial similarity matrices for pairwise comparisons between groups. (C) Cosine similarity between five states and seven functional networks of the brain.

The CAP analysis identified five spatiotemporally distinct brain network states across all groups, demonstrating the spatial configurations with high within‐group consistency (diagonal similarity, Figure 2A,B). The five states were as follows: State 1 (limbic network dominated), State 2 (dorsal attention network (DAN) and CEN dominated), State 3 (DMN and CEN dominated), State 4 (somatomotor network and ventral attention network dominated), and State 5 (sensorimotor network, DAN, and visual network dominated). Considering that the spatial patterns of the five CAP states were associated with well‐known functional networks (Lee et al. 2024), we calculated the cosine similarity between them (Table S1 and Figure 2C). State 1 and State 2 exhibited strong bi‐polarity between DAN and CEN, while the limbic network displayed an inverse pattern. Bi‐polarity refers to the positive and negative cosine similarity between each CAP and the different functional networks. Positive similarity indicated that the spatial activation pattern of the CAP state was consistent with the direction of the functional network template, suggesting that the network was activated or involved in the state. Negative similarity indicated that the spatial activation pattern of the CAP state was opposite to the direction of the functional network template, suggesting that the network was inhibited or antagonized in the state. State 3 shows the activation of CEN and DMN. State 4 shows the activation of the somatomotor network and ventral attention network. State 5 is more likely to show the activation of DAN, sensorimotor, and visual networks. Additionally, a strong negative correlation was observed between State 1 and State 2 (*r* = −0.99, *p* < 0.0001), which underscored their high anticorrelation (Figure S2).

### 3.2 Temporal Dynamic Differences

**FIGURE 3:**
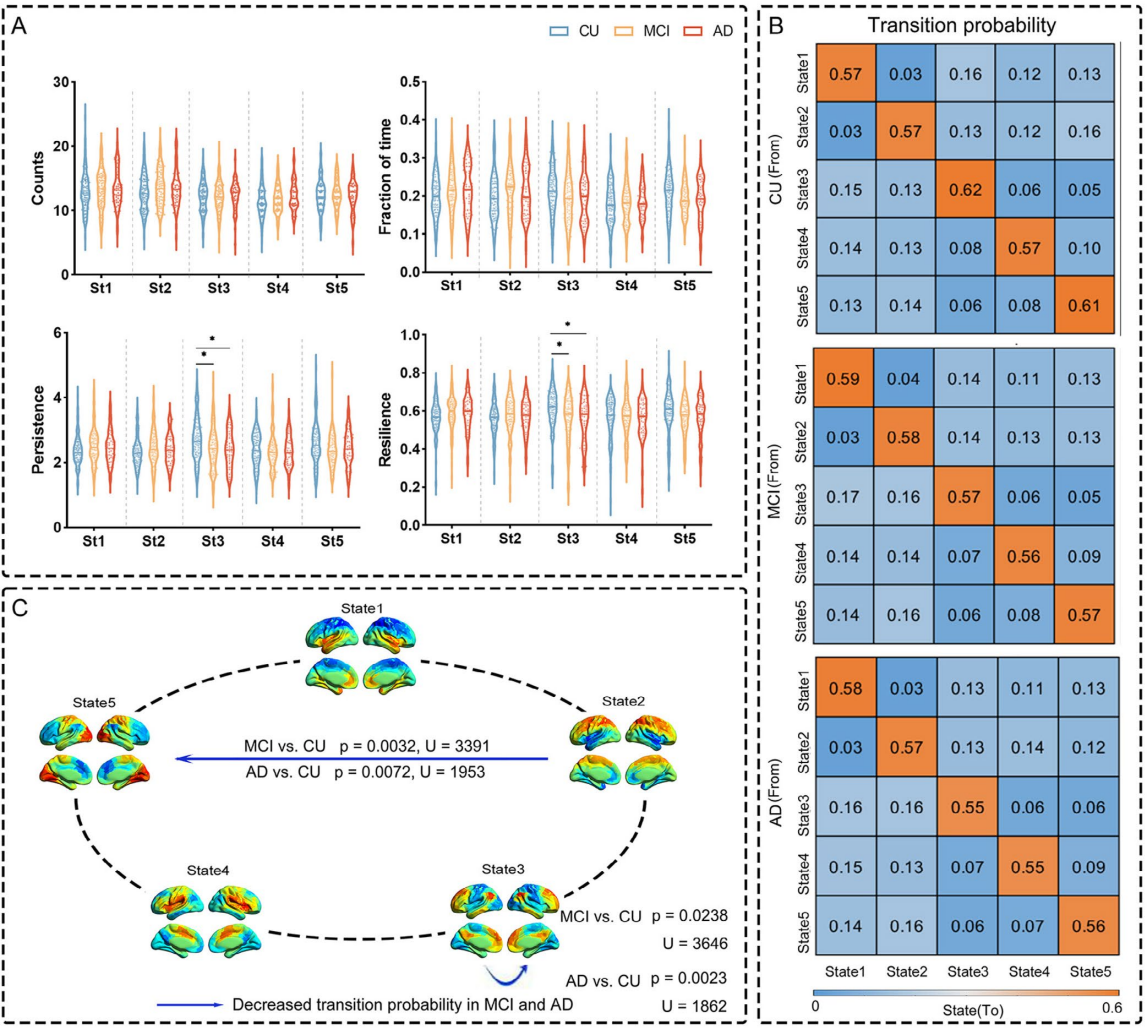
Temporal dynamics of CAP. (A) Statistical difference of CAP states among CU, MCI, and AD groups. (B) State transition probability matrices for each group. (C) Visualizes significant differences in state transitions among groups. The FDR‐correction with *p* < 0.05 was performed to estimate the significance, and the total number of tests was 25.

Individual‐level dynamic metrics, including the counts, fraction of time, persistence, resilience, and transition probability, were calculated for each CAP state, and the between‐group differences were analyzed using the Kruskal–Wallis test (FDR‐correction with *p* < 0.05; total number of tests = 5). Notably, State 3 exhibited significantly reduced persistence and resilience in both MCI and AD compared to CU (Figure 3A). Transition probability matrices revealed significant between‐group differences in the state dynamics. Specifically, compared to CU, individuals with MCI and AD showed reduced transitions from State 2 to State 5, and decreased self‐transitions in State 3 (Figure 3B,C).

### 3.3 Graph Theoretical Differences

**FIGURE 4:**
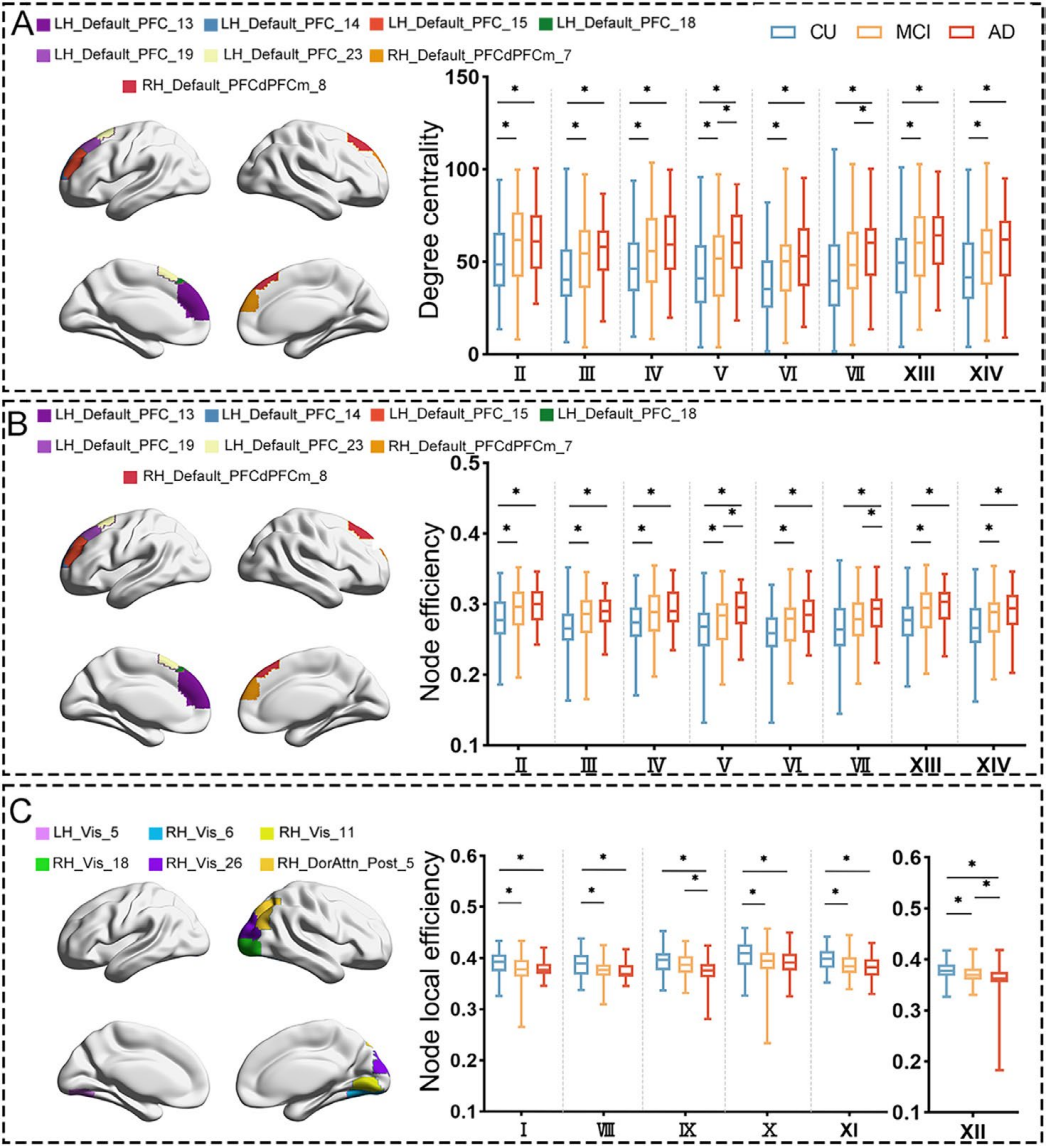
Node‐level topological differences in graph analysis. (A) Significant differences in the degree of centrality of different ROIs. (B) Significant differences in nodal efficiency of different ROIs. (C) Significant differences in nodal local efficiency of different ROIs. FDR was used for multiple comparisons with *p* < 0.05, and the total number of tests was 400. I = LH_VIS_5; II = LH_Default_PFC_13; III = LH_Default_PFC_14; IV = LH_Default_PFC_15; V = LH_Default_PFC_18; VI = LH_Default_PFC_19; VII = LH_Default_PFC_23; VII = RH_VIS_6; IX = RH_VIS_11; X = RH_VIS_18; XI = RH_VIS_26; XII = RH_DorsAttn_Post_5; XIII = RH_Default_PFCdPFCm_7; and XIV = RH_Default_PFCdPFCm_7.

To further investigate the system‐level mechanisms underlying the dynamic differences identified in CAP analysis, graph theory analysis was performed to quantify network topological properties associated with cognitive states. Graph theoretical analysis identified diagnosis‐related regions that were distributed in the DMN, DAN, and visual network (Table 2, Figure 4). Compared to CU, individuals with MCI and AD showed significant differences in the right superior frontal gyrus, right superior medial frontal gyrus, right lingual gyrus, and right fusiform gyrus. Specifically, compared to CU, both MCI and AD exhibited significantly higher degree centrality and node efficiency in the upper part of the left medial superior frontal gyrus (LH_Default_PFC_13) (degree centrality: MCI vs. CU, *p* = 0.002, *U* = 3317; AD vs. CU, *p* < 0.001, *U* = 1717. Node efficiency: MCI vs. CU, *p* = 0.002, *U* = 3355; AD vs. CU, *p* < 0.001, *U* = 1718). MCI and AD individuals also showed significantly higher degree centrality and node efficiency in the upper part of the right medial superior frontal gyrus (RH_Default_PFCdPFCm_7) compared to CU (Degree centrality: MCI vs. CU, *p* = 0.001, *U* = 3298; AD vs. CU, *p* < 0.001, *U* = 1655. Node efficiency: MCI vs. CU, *p* < 0.001, *U* = 3254; AD vs. CU, *p* < 0.001, *U* = 1622). Furthermore, relative to CU, both MCI and AD groups demonstrated significantly increased degree centrality and node efficiency in the superior frontal gyrus (Left: LH_Default_PFC_14/15/19; Right: RH_Default_PFC_PFCdPFCm_8) (Table 2, Figure 4A,B). In contrast, both MCI and AD exhibited significantly lower local efficiency in the left lingual gyrus (LH_VIS_5) compared to CU (MCI vs. CU: *p* < 0.001, *U* = 3664; AD vs. CU: *p* < 0.001, *U* = 1416). Similarly, the node local efficiency in the right fusiform gyrus (RH_VIS_6) also showed a significant decline in individuals with MCI and AD compared to CU (MCI vs. CU: *p* = 0.003, *U* = 3780; AD vs. CU: *p* < 0.001, *U* = 1372). However, node local efficiency in the right middle occipital gyrus (RH_VIS_18/26) was significantly decreased in MCI and AD compared to CU (Table 2, Figure 4C).

**TABLE 2:** Statistical analysis of different graph theoretical parameters between CU, MCI, and AD groups.

ROI name	MCI vs. CU	AD vs. CU	AD vs. MCI
LH_Default_PFC_13	*p* = 0.002,^a^ *U* = 3317, |*d*| = 0.263	*p* < 0.001,^a^ *U* = 1717, |*d*| = 0.352	*p* = 0.570, *U* = 2249, |*d*| = 0.057
LH_Default_PFC_14	*p* < 0.001,^a^ *U* = 3172, |*d*| = 0.295	*p* < 0.001,^a^ *U* = 1540, |*d*| = 0.419	*p* = 0.442, *U* = 2200, |*d*| = 0.078
LH_Default_PFC_15	*p* = 0.004,^a^ *U* = 3406, |*d*| = 0.243	*p* < 0.001,^a^ *U* = 1631, |*d*| = 0.385	*p* = 0.212, *U* = 2086, |*d*| = 0.125
LH_Default_PFC_18	*p* = 0.031,^a^ *U* = 3682, |*d*| = 0.182	*p* < 0.001,^a^ *U* = 1402, |*d*| = 0.471	*p* = 0.006,^a^ *U* = 1731, |*d*| = 0.274
LH_Default_PFC_19	*p* = 0.002,^a^ *U* = 3345, |*d*| = 0.257	*p* < 0.001,^a^ *U* = 1548, |*d*| = 0.416	*p* = 0.121, *U* = 2014, |*d*| = 0.156
LH_Default_PFC_23	*p* = 0.060, *U* = 3788, |*d*| = 0.158	*p* < 0.001,^a^ *U* = 1650, |*d*| = 0.377	*p* = 0.034,^a^ *U* = 1879, |*d*| = 0.212
RH_Default_PFCdPFCm_7	*p* = 0.001,^a^ *U* = 3298, |*d*| = 0.267	*p* < 0.001,^a^ *U* = 1655, |*d*| = 0.376	*p* = 0.389, *U* = 2178, |*d*| = 0.087
RH_Default_PFCdPFCm_8	*p* = 0.009,^a^ *U* = 3508, |*d*| = 0.220	*p* < 0.001,^a^ *U* = 1638, |*d*| = 0.382	*p* = 0.146, *U* = 2036, |*d*| = 0.146
LH_Default_PFC_13	*p* = 0.002,^a^ *U* = 3355, |*d*| = 0.254	*p* < 0.001,^a^ *U* = 1718, |*d*| = 0.352	*p* = 0.542, *U* = 2238, |*d*| = 0.062
LH_Default_PFC_14	*p* < 0.001,^a^ *U* = 3215, |*d*| = 0.286	*p* < 0.001,^a^ *U* = 1532, |*d*| = 0.422	*p* = 0.367, *U* = 2168, |*d*| = 0.091
LH_Default_PFC_15	*p* = 0.006,^a^ *U* = 3459, |*d*| = 0.231	*p* < 0.001,^a^ *U* = 1655, |*d*| = 0.376	*p* = 0.211, *U* = 2085, |*d*| = 0.126
LH_Default_PFC_18	*p* = 0.027,^a^ *U* = 3662, |*d*| = 0.186	*p* < 0.001,^a^ *U* = 1401, |*d*| = 0.471	*p* = 0.006,^a^ *U* = 1724, |*d*| = 0.277
LH_Default_PFC_19	*p* = 0.002,^a^ *U* = 3356, |*d*| = 0.254	*p* < 0.001,^a^ *U* = 1563, |*d*| = 0.410	*p* = 0.119, *U* = 2011, |*d*| = 0.157
LH_Default_PFC_23	*p* = 0.063, *U* = 3797, |*d*| = 0.156	*p* < 0.001,^a^ *U* = 1641, |*d*| = 0.381	*p* = 0.023,^a^ *U* = 1840, |*d*| = 0.229
RH_Default_PFCdPFCm_7	*p* < 0.001,^a^ *U* = 3254, |*d*| = 0.277	*p* < 0.001,^a^ *U* = 1622, |*d*| = 0.388	*p* = 0.339, *U* = 2155, |*d*| = 0.096
RH_Default_PFCdPFCm_8	*p* = 0.009,^a^ *U* = 3508, |*d*| = 0.220	*p* < 0.001,^a^ *U* = 1660, |*d*| = 0.374	*p* = 0.146, *U* = 2036, |*d*| = 0.146
LH_VIS_5	*p* = 0.001,^a^ *U* = 3664, |*d*| = 0.186	*p* = 0.001,^a^ *U* = 1416, |*d*| = 0.466	*p* = 0.718, *U* = 2067, |*d*| = 0.133
RH_VIS_6	*p* = 0.003,^a^ *U* = 3780, |*d*| = 0.160	*p* = 0.001,^a^ *U* = 1372, |*d*| = 0.482	*p* = 0.177, *U* = 1842, |*d*| = 0.228
RH_VIS_11	*p* = 0.069, *U* = 4256, |*d*| = 0.054	*p* < 0.001,^a^ *U* = 1280, |*d*| = 0.517	*p* = 0.004,^a^ *U* = 1492, |*d*| = 0.374
RH_VIS_18	*p* < 0.001,^a^ *U* = 3544, |*d*| = 0.212	*p* = 0.005,^a^ *U* = 1509, |*d*| = 0.431	*p* = 0.737, *U* = 2073, |*d*| = 0.131
RH_VIS_26	*p* = 0.001,^a^ *U* = 3691, |*d*| = 0.180	*p* = 0.001,^a^ *U* = 1425, |*d*| = 0.462	*p* = 0.411, *U* = 1962, |*d*| = 0.177
RH_DorsAttn_Post_5	*p* = 0.010,^a^ *U* = 3942, |*d*| = 0.124	*p* < 0.001,^a^ *U* = 1245, |*d*| = 0.530	*p* = 0.022,^a^ *U* = 1631, |*d*| = 0.316

*Note:* |*d*| represents the cliff delta. The total number of tests was 400.

^a^ FDR correction with *p* < 0.05.

Compared to CU, significantly increased degree centrality and node efficiency were observed in the upper left medial frontal gyrus (LH_Default_PFC_18) in individuals with MCI and AD. Notably, AD exhibited a significantly increased degree centrality and node efficiency in the region compared to MCI. Furthermore, local efficiency in the right middle occipital gyrus (RH_DorAttn_Post_5) showed significant reductions in both MCI (*p* = 0.010, *U* = 3942) and AD (*p* < 0.001, *U* = 1245) compared to CU. Moreover, AD displayed significantly lower local efficiency in the region than that of MCI (*p* = 0.022, *U* = 1631).

We also observed the distinct alterations in graph theory parameters specific to MCI and AD groups. Notably, AD manifested significantly increased degree centrality and node efficiency in the left superior frontal gyrus (LH_Default_PFC_23) compared to CU (degree centrality: *p* < 0.001, *U* = 1650; Node efficiency: *p* < 0.001, *U* = 1641). However, these metrics showed no significant differences in MCI relative to CU. Furthermore, compared to CU, the AD group had a significantly reduced local efficiency in the right lingual gyrus (RH_VIS_11) (*p* = 0.0342, *U* = 1879), while the MCI group did not show a significant difference from CU.

## 4 Discussion

This study combined the CAP method and graph theoretical analysis to investigate the abnormal state transitions and the corresponding topological characteristics in distinct stages of AD. We observed that State 3 exhibited significantly reduced persistence and resilience in both MCI and AD compared to CU. Individuals with MCI and AD showed reduced transition probability from State 2 to State 5 and decreased self‐transitions in State 3. Additionally, graph theoretical analysis revealed abnormal topological regions widely distributed in DMN, visual network, and DAN, which corresponded well to the CAP analysis results. This study systematically analyzed the hierarchical disruption of functional systems in individuals with MCI and AD, providing a multiscale framework to link dynamic state instability, static network reorganization, and clinical progression.

The CAP analysis revealed five spatially conserved brain states across CU, MCI, and AD groups, underscoring that core dynamic brain repertoires remain preserved despite neurodegeneration (Damoiseaux et al. 2006). Through spatial similarity mapping with Yeo's 7‐network parcellation, these states exhibited distinct functional roles. For instance, State 1 had a strong preponderance of cosine similarity in the limbic network, suggesting its pivotal role in integrating emotional experiences with episodic memory (Pessoa 2008). Compared to CU, State 3 was characterized by a significant reduction in persistence and resilience in both MCI and AD. State 3 is characterized by activation of DMN and CEN, alongside suppression of the sensorimotor network. DMN supports the autobiographical memory retrieval and self‐referential thought (Ronde et al. 2024; Smallwood et al. 2021), while CEN mediates the goal decomposition and planning (Bigliassi et al. 2025). Their transient coordination facilitates future scenario simulation, like planning, based on prior experiences (Vatansever et al. 2017), suggesting the important role of State 3 in dynamically allocating internal and external cognitive resources to balance introspection and goal‐directed adaptation. The decreased persistence and resilience in State 3 indicated the progressive deterioration of dynamic coordination capacity between DMN and CEN in individuals with MCI and AD. Additionally, AD pathology, specifically amyloid‐β deposition and tau neurofibrillary tangles, disproportionately affects regions of DMN, including the posterior cingulate cortex and medial prefrontal cortex (Greicius et al. 2003), thereby impairing DMN and CEN synchronization. This disruption in coordination may compromise patients' ability to sustain complex cognitive states, manifested as reduced duration (persistence) and stability (resilience) of State 3, directly impairing patients' daily functioning. For instance, autobiographical memory retrieval and prospective planning rely on dynamic integration between DMN and CEN (Smallwood et al. 2021).

Transition probability matrices revealed significantly decreased transitions from State 2 (CEN and DAN dominated) to State 5 (DAN, sensorimotor, and visual networks dominated) in both MCI and AD, alongside reduced self‐transition frequency in State 3. While both State 2 and 5 involve DAN, their functional emphases differ. State 2 supported working memory and spatial attention (Shine et al. 2023), whereas State 5 mediated visual environmental monitoring (Corbetta and Shulman 2002). The reduced State 2‐to‐State 5 transitions may indicate impaired flexibility in reallocating attentional resources according to task demands, reflecting emerging cognitive rigidity. This phenomenon aligns with previous findings regarding between‐network functional segregation in individuals with AD (Zhou et al. 2010), characterized by attenuated antagonistic relationships between DMN and task‐positive networks. Furthermore, the reduced self‐transitions in State 3 signified the diminished connectivity redundancy within DMN nodes (Li et al. 2024), compromising patients' ability to sustain stable introspective states and prompting frequent shifts to alternative network configurations. These two aberrant transition patterns exacerbated the clinical phenotypes in individuals with MCI and AD. For instance, visuospatial deficits in AD (e.g., spatial disorientation) could be associated with hypoactivation of State 5, while attentional control impairments (e., increased distractibility) may stem from inefficient State 2‐to‐State 5 transitions (Ptak 2012).

Building on the CAP findings of disrupted network coordination in AD, graph theoretical analysis further revealed spatiotemporal signatures of multilevel network reorganization from a static topological perspective. Specific prefrontal DMN nodes (e.g., LH_Default_PFC_13–15) exhibited significantly increased degree centrality and nodal efficiency in both MCI and AD compared to CU, with no difference between MCI and AD. This result reflected compensatory hyperconnectivity in prefrontal regions triggered by posterior DMN disconnection (e.g., posterior cingulate cortex) during early MCI stages, followed by a plateau phase of compensation in AD (Tijms et al. 2013; Buckner et al. 2009; Sperling et al. 2009). Moreover, gradual changes in topological metrics were observed from CU to MCI to AD. Progressively increased degree centrality and node efficiency in the left prefrontal cortex (e.g., LH_Default_PFC_18) across the CU–MCI–AD continuum reflected heightened integrative roles of prefrontal regions to compensate for progressive degradation of posterior DMN hubs (Buckner et al. 2009). Declining local efficiency in the RH_DorAttn_Post_5 indicated gradual disintegration of DAN with disease progression, which may underlie deficits in spatial attention (Corbetta and Shulman 2002). These gradient‐based metrics quantitatively capture the continuous network evolution from preclinical to dementia stages (Li et al. 2024), offering a tool for dynamically monitoring disease progression and evaluating therapeutic interventions (Preti et al. 2017).

The AD‐specific alterations revealed that compared to CU and MCI, AD exhibited unique reduced local efficiency in the right lingual gyrus (RH_VIS_11), which was linked to tau pathology spreading to visual‐associated white matter tracts (e.g., inferior longitudinal fasciculus) in AD (Li et al. 2024). Increased degree centrality and node efficiency in the left prefrontal cortex (e.g., LH_Default_PFC_23) indicated forced hub reconfiguration to integrate residual network resources amid widespread neurodegeneration (Seeley et al. 2009). These AD‐specific signatures marked the irreversible progression into clinical dementia, indicating exhaustion of compensatory plasticity (Zhou et al. 2010), which could aid clinical staging by distinguishing late‐stage AD from earlier phases.

By combining the CAP and graph theoretical analysis, the study provides a multiscale perspective on the hierarchical collapse of functional networks in AD. The dynamic instability observed in CAP states, particularly the reduced persistence and resilience of State 3 (DMN and CEN coordination), aligns with static topological alterations in the degradation of prefrontal and parietal hubs, collectively reflecting the interplay between compensatory adaptation and functional disintegration. For instance, the diminished stability of State 3 in individuals with MCI and AD, characterized by transient DMN and CEN interactions, may stem from dual effects of posterior hub atrophy in DMN (Greicius et al. 2003) and compensatory hyperconnectivity in prefrontal regions. Graph theoretical findings revealed increased degree centrality and nodal efficiency in the prefrontal nodes of DMN (e.g., LH_Default_PFC_13–15) across both MCI and AD, suggesting a resource reallocation strategy to offset posterior network deficits (Buckner et al. 2009). However, this compensatory enhancement appears insufficient to sustain dynamic coordination between DMN and CEN, as evidenced by State 3's reduced persistence. This dissociation implies that while static network metrics capture localized compensatory efforts, dynamic metrics reflect the failure of these adaptations to stabilize high‐order cognitive states critical for memory and planning (Smallwood et al. 2021). Additionally, the reduced transitions from State 2 (CEN and DAN dominated) to State 5 (DAN and visual network dominated) in AD may be mechanistically linked to decreased local efficiency in RH_DorsAttn_Post_5. Graph theoretical analysis identified the progressive declines of nodal local efficiency in the RH_DorsAttn_Post_5, indicating the impaired intra‐network communication. Such localized inefficiency likely disrupts the rapid reconfiguration of attentional resources required for visuospatial monitoring (Corbetta and Shulman 2002), thereby limiting the flexible state transitions. These findings underscore how static network disintegration constrains dynamic adaptability, a hallmark of cognitive rigidity in AD (Zhou et al. 2010). Moreover, by combining gene expression and dynamic functional networks analysis, researchers demonstrated the close relationships between genetic variations and dynamic functional features in AD (Rubido et al. 2024). Dynamic functional connectivity in resting‐state fMRI could be an effective biomarker for early diagnosis and monitoring of AD progression (Tessadori et al. 2025). These studies indicated the necessity of investigating the dynamic characteristics of brain function in AD, which would promote clinical diagnosis and treatment decisions for patients.

## 5 Limitations

This study has several limitations that warrant consideration. First, the cross‐sectional design precludes causal inferences about the observed dynamic and static network alterations. Longitudinal studies are needed to determine whether these changes precede clinical symptoms or result from neurodegenerative progression. Second, heterogeneous medication use (e.g., acetylcholinesterase inhibitors and memantine) among patients, particularly in individuals with AD, may confound observed network alterations. Variations in regimens could influence graph metrics, limiting definitive attribution of differences solely to disease pathology.

## 6 Conclusions

This study employed CAP and graph theoretical analysis to investigate the dynamic brain state organization and static network topology alterations in individuals with MCI and AD. Both MCI and AD exhibited reduced persistence and resilience of State 3, indicating impaired coordination between DMN and CEN, along with disrupted state transitions reflecting cognitive inflexibility. Concurrently, static network reorganization featured compensatory hyperconnectivity within prefrontal hubs and progressive degradation of posterior and attention networks, with AD‐specific disruptions marking irreversible disease progression. Critically, the combined evidence from dynamic instability and static topology alterations indicated disordered switching between different brain functional systems in individuals with AD. Our findings elucidate the layered disruption of distinct functional systems, enhancing the understanding of network‐level neurodegenerative mechanisms in AD.

## Funding

This study is supported by grants from the China MOST2030 Brain Project (grant number: 2022ZD0208500), the National Natural Science Foundation of China (grant numbers: NSFC62401106 and NSFC62171101), and the Sichuan Science and Technology Program (grant number: 2024NSFSC1661).

## Conflicts of Interest

The authors declare no conflicts of interest.

## Supporting information


**Data S1:** Supporting Information.

## Data Availability

The neuroimaging dataset in the study was provided by OASIS OASIS‐3: Longitudinal Multimodal Neuroimaging: Principal Investigators: T. Benzinger, D. Marcus, and J. Morris; NIH P30 AG066444, P50 AG00561, P30 NS09857781, P01 AG026276, P01 AG003991, R01 AG043434, UL1 TR000448, and R01 EB009352. AV‐45 doses were provided by Avid Radiopharmaceuticals, a wholly owned subsidiary of Eli Lilly.
