# Supplementary material for: Dynamic Alterations of Functional Systems in Alzheimer's Disease: A Co‐Activation Pattern Analysis
Source: Hum Brain Mapp. 2026 Mar 26;47(5):e70509. doi: 10.1002/hbm.70509 (PMC13081692; doi:10.1002/hbm.70509)
Supplement: Supplementary file 1 — Data S1: Supporting Information. [file HBM-47-e70509-s001.docx]

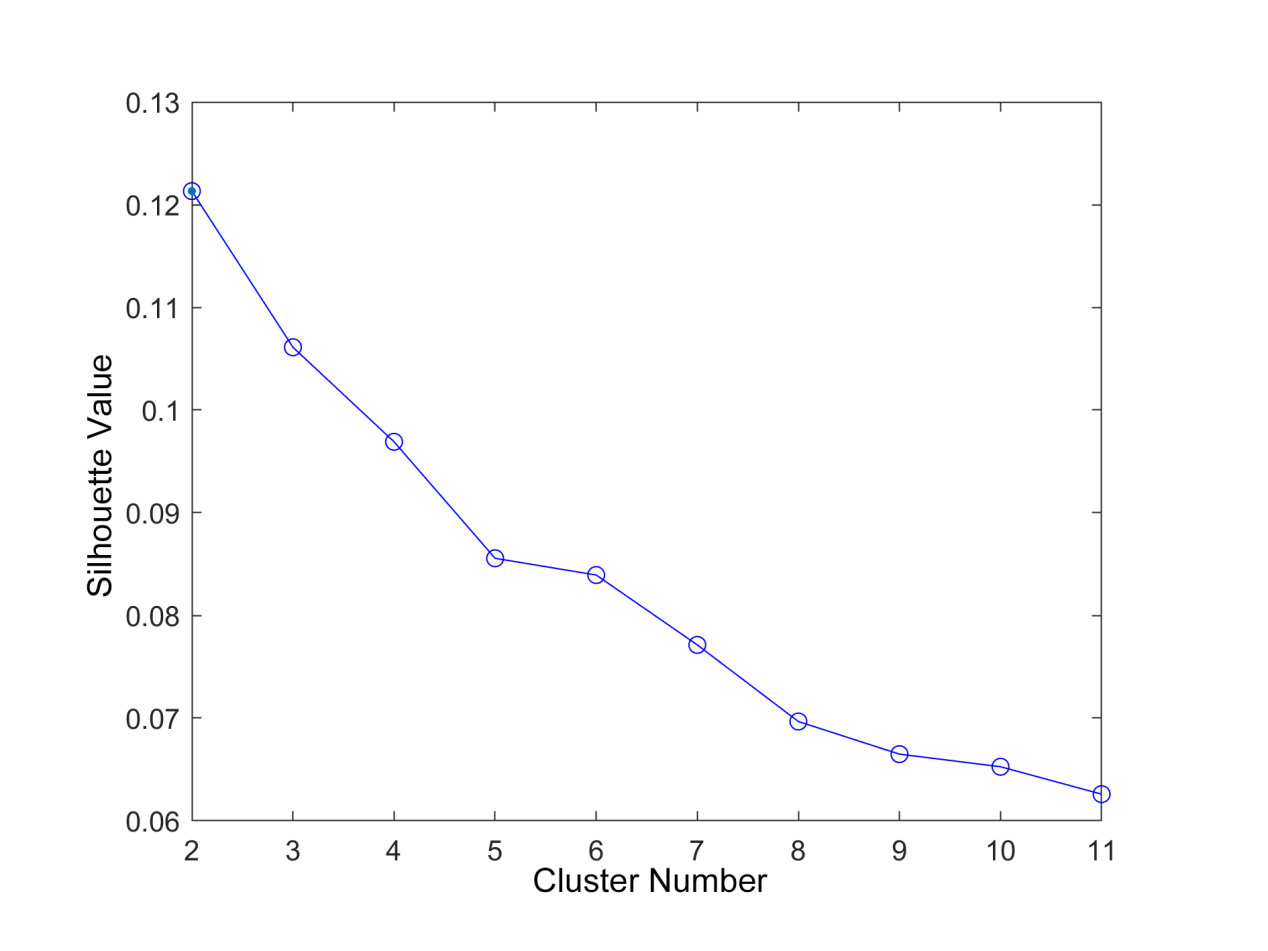


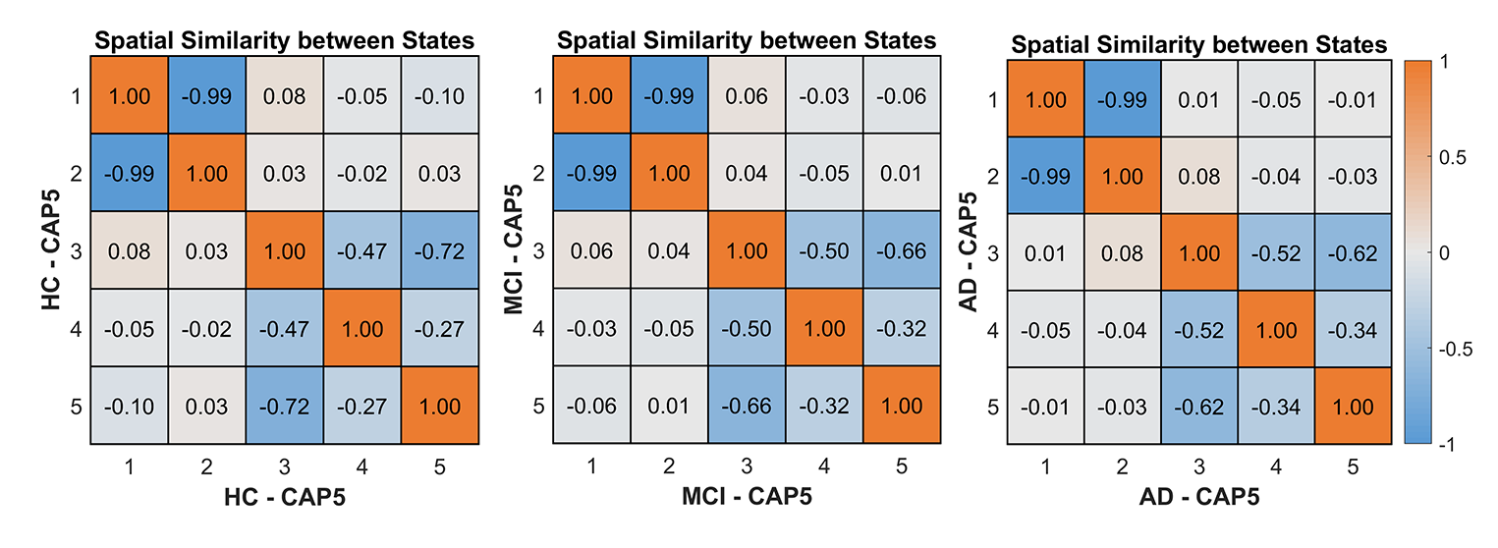
**Fig. S1.** The relationship between the contour Silhouette value and cluster number.

**Fig. S2.** Spatial similarity among different states within group.

**Table S1.** Cosine similarity between five states and seven functional networks of brain.

| Corresponding FN | State1 | State2 | State3 | State4 | State5 |
| --- | --- | --- | --- | --- | --- |
| Visual | -0.1572 | -0.2335 | -0.9146 | -0.9642 | 0.9767 |
| Somatomotor | -0.5038 | 0.2971 | -0.9758 | 0.9082 | 0.7781 |
| Dorsal Attention | -0.8690 | 0.8447 | -0.7323 | 0.2303 | 0.8255 |
| Salience/Ventral Attention | -0.1832 | -0.0384 | -0.5770 | 0.9177 | -0.6975 |
| Limbic | 0.6920 | -0.6530 | 0.5520 | -0.8985 | 0.7260 |
| Central Executive | -0.8069 | 0.8026 | 0.8776 | 0.1146 | -0.8356 |
| Default | 0.1864 | -0.2342 | 0.7679 | -0.4569 | -0.6236 |

Note: FN = functional networks.


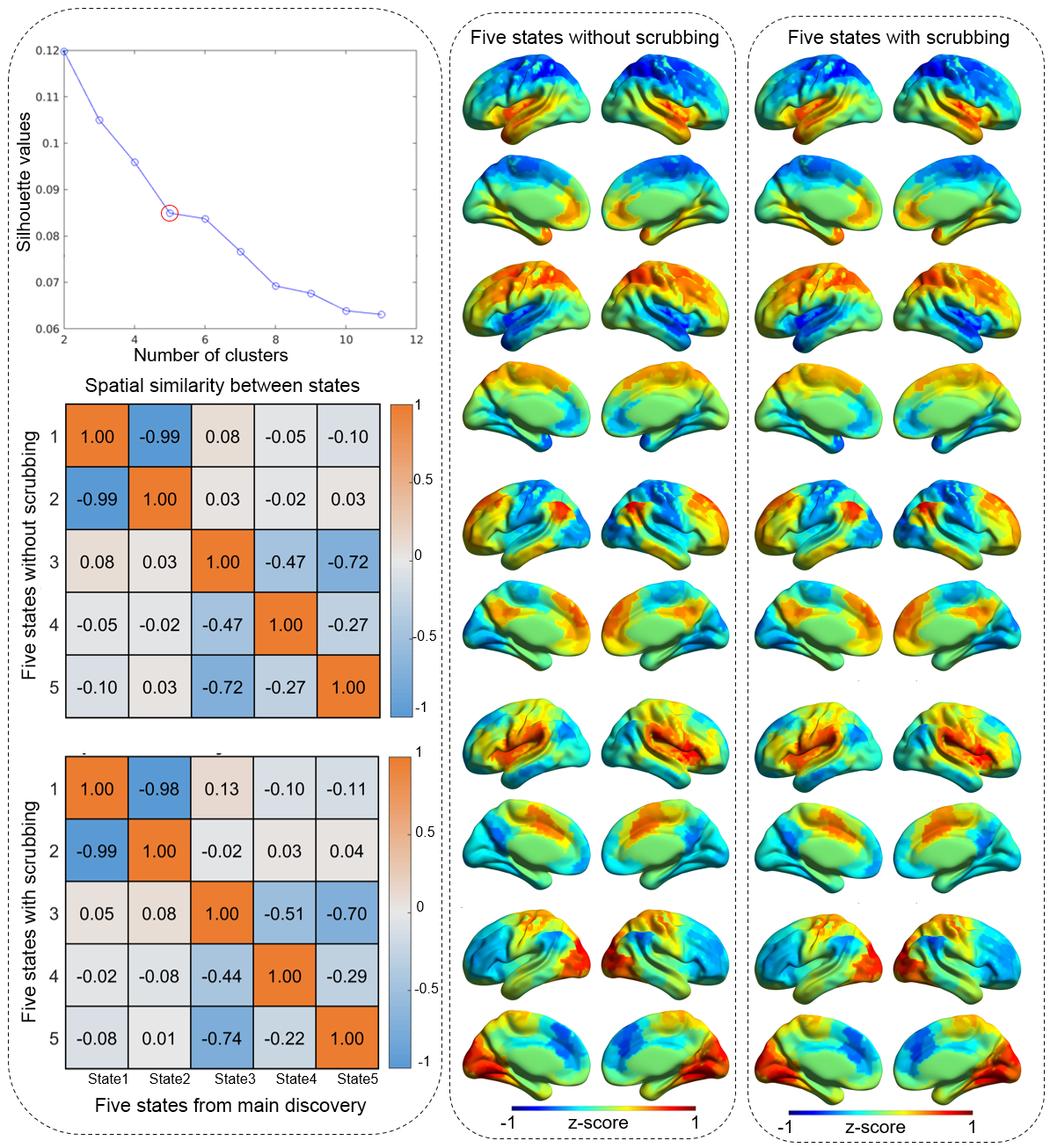


Figure S3. The reproducible analysis of head motion scrubbing regressors. The left side shows the optimal number of clusters and the spatial similarity between different states. The middle section displays the state distribution patterns in the main manuscript. The right side shows the state distribution patterns of reproducibility analysis.


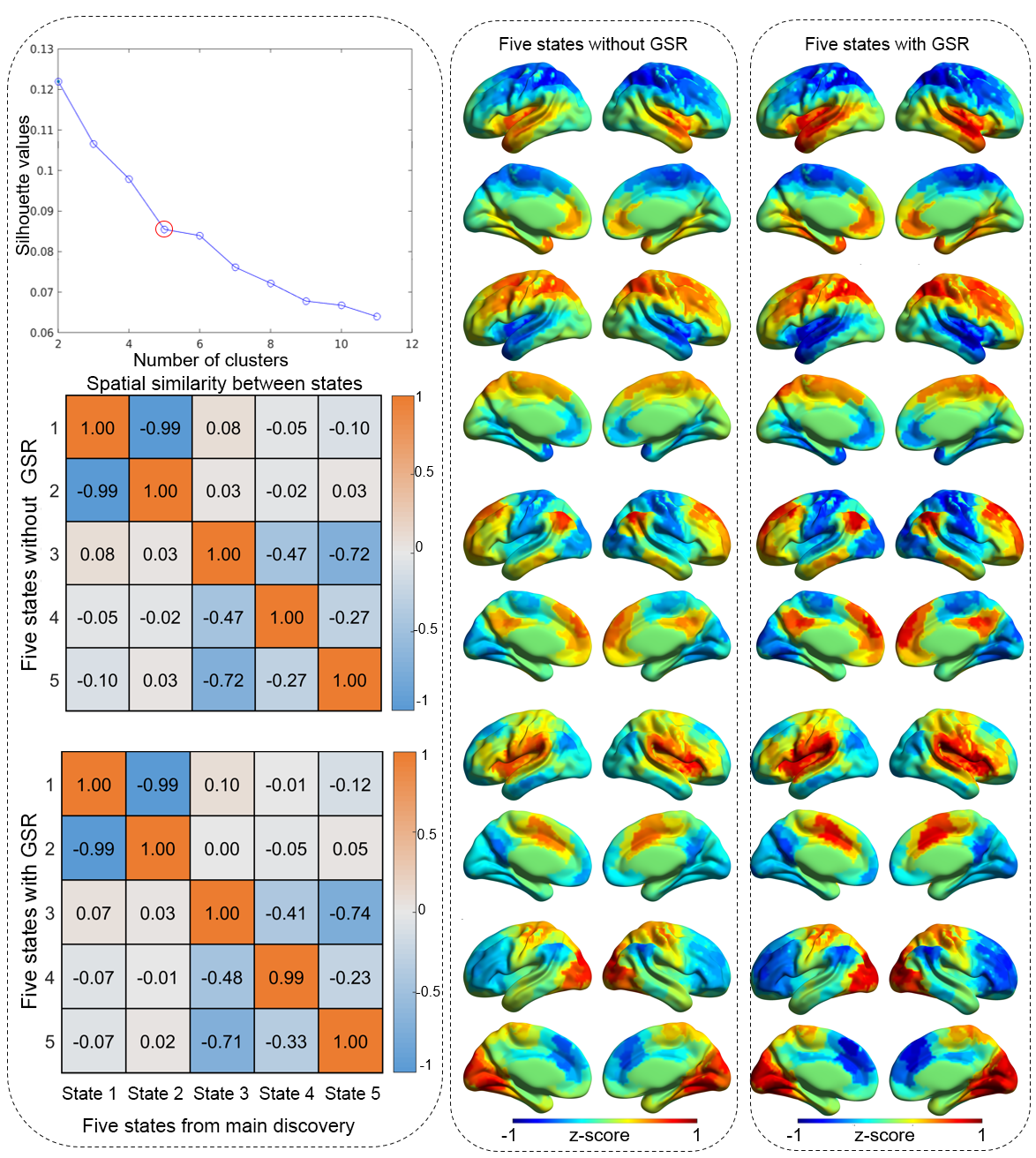


Figure S4. The reproducible analysis of global signal regression. The left side shows the optimal number of clusters and the spatial similarity between different states. The middle section displays the state distribution patterns in the main manuscript. The right side shows the state distribution patterns of reproducibility analysis.


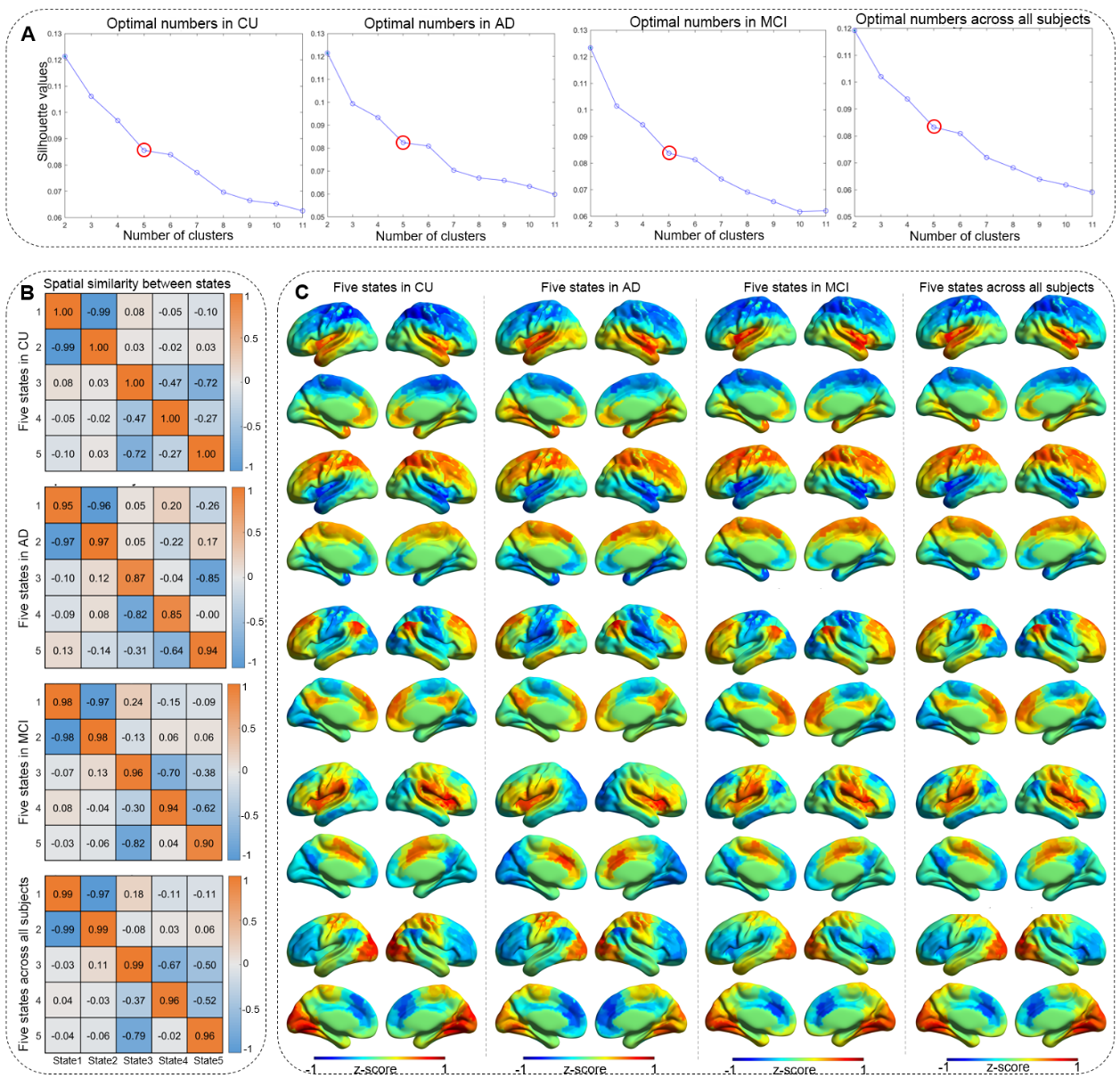


Figure S5. The reproducible analysis of clustering states across different groups. The matrix of left side shows spatial similarity between different states corresponding to all subjects, only patients and CU individuals. The right side exhibits the spatial distribution pattern maps of five states across all subjects, only patients and CU individuals.


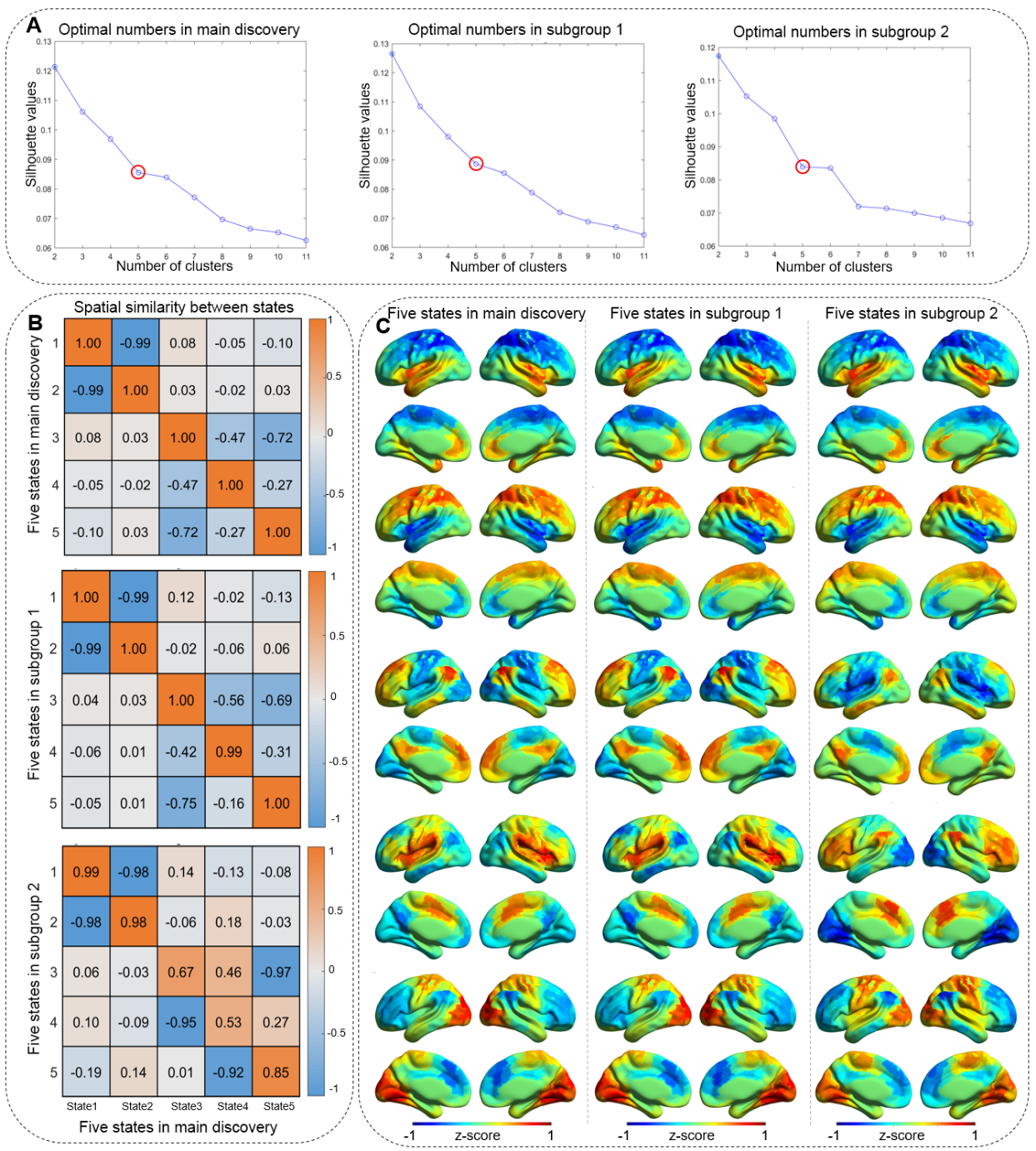


Figure S6. The reproducible analysis of CAPs using split-half reliability method. The matrix of left side shows spatial similarity between different states corresponding to different groups. The right side exhibits the spatial distribution pattern maps of five states.


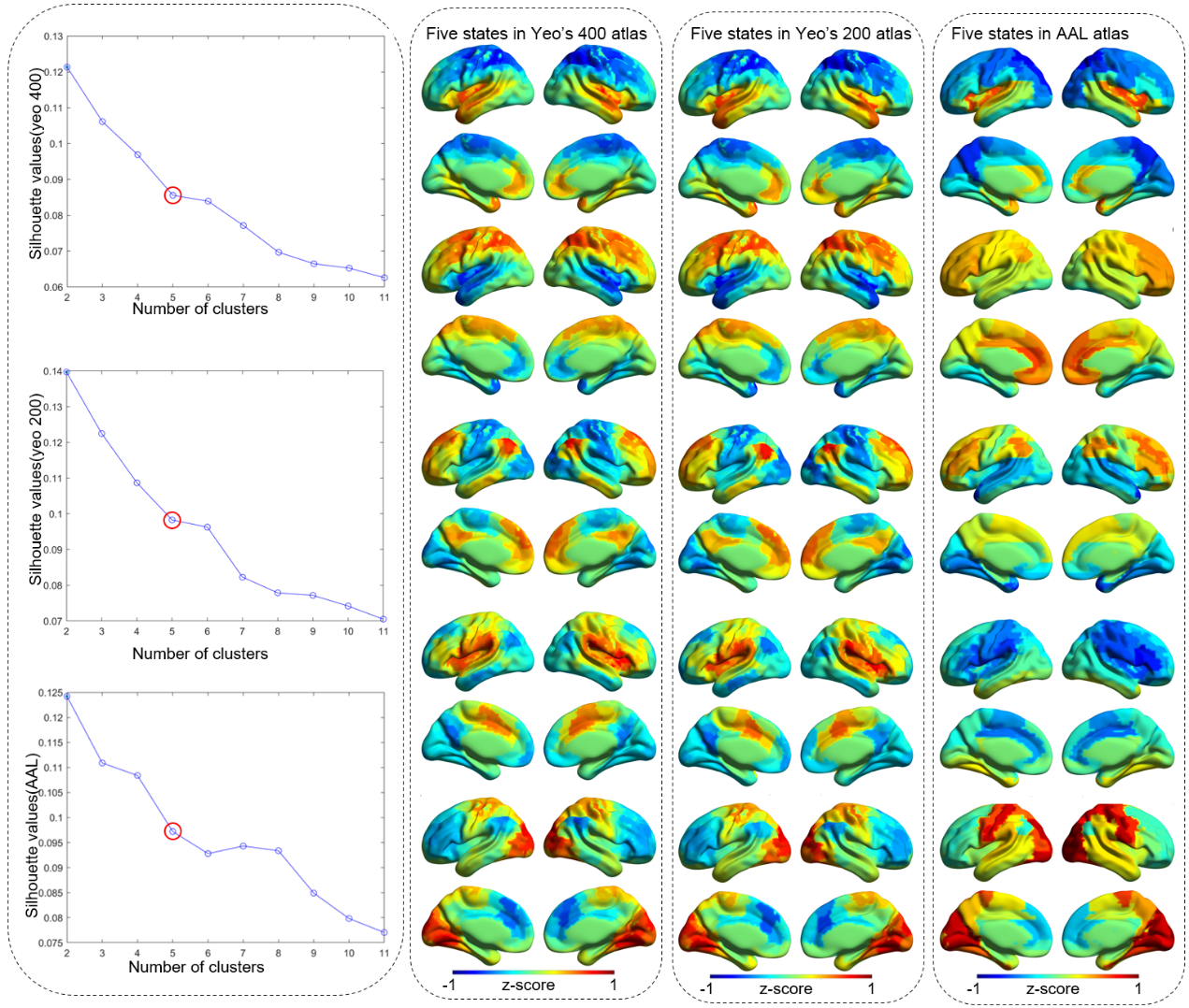


Figure S7. The reproducible analysis for alternative parcellation schemes. The left side shows the optimal number of clusters. The right side shows the different states based on Yeo 200/400 and AAL atlas.


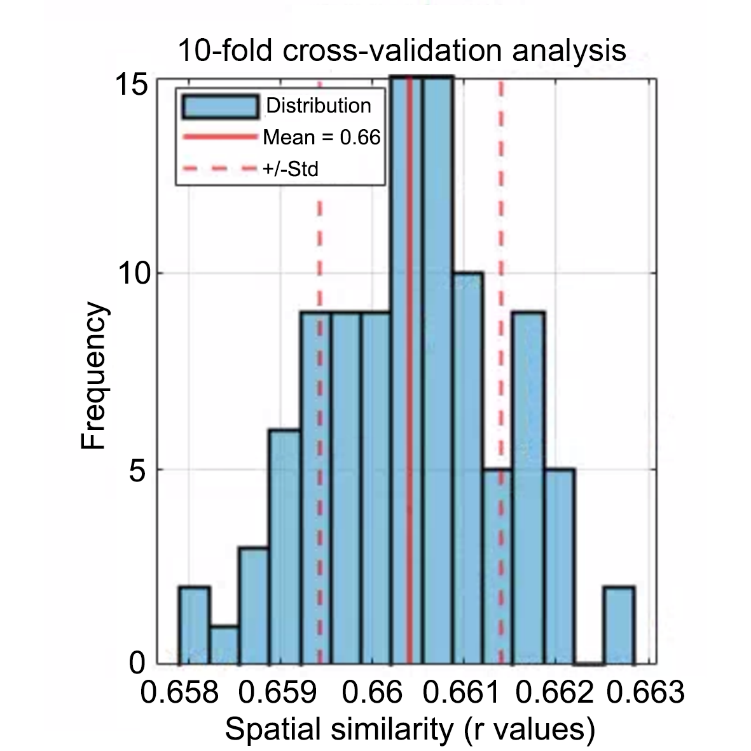


Figure S8. The reproducible analysis of CAPs using 10-fold cross-validation analysis. The horizontal axis represents the spatial similarity between states from reproducible analysis and main manuscript. The vertical axis represents the frequency of occurrence (n = 100).


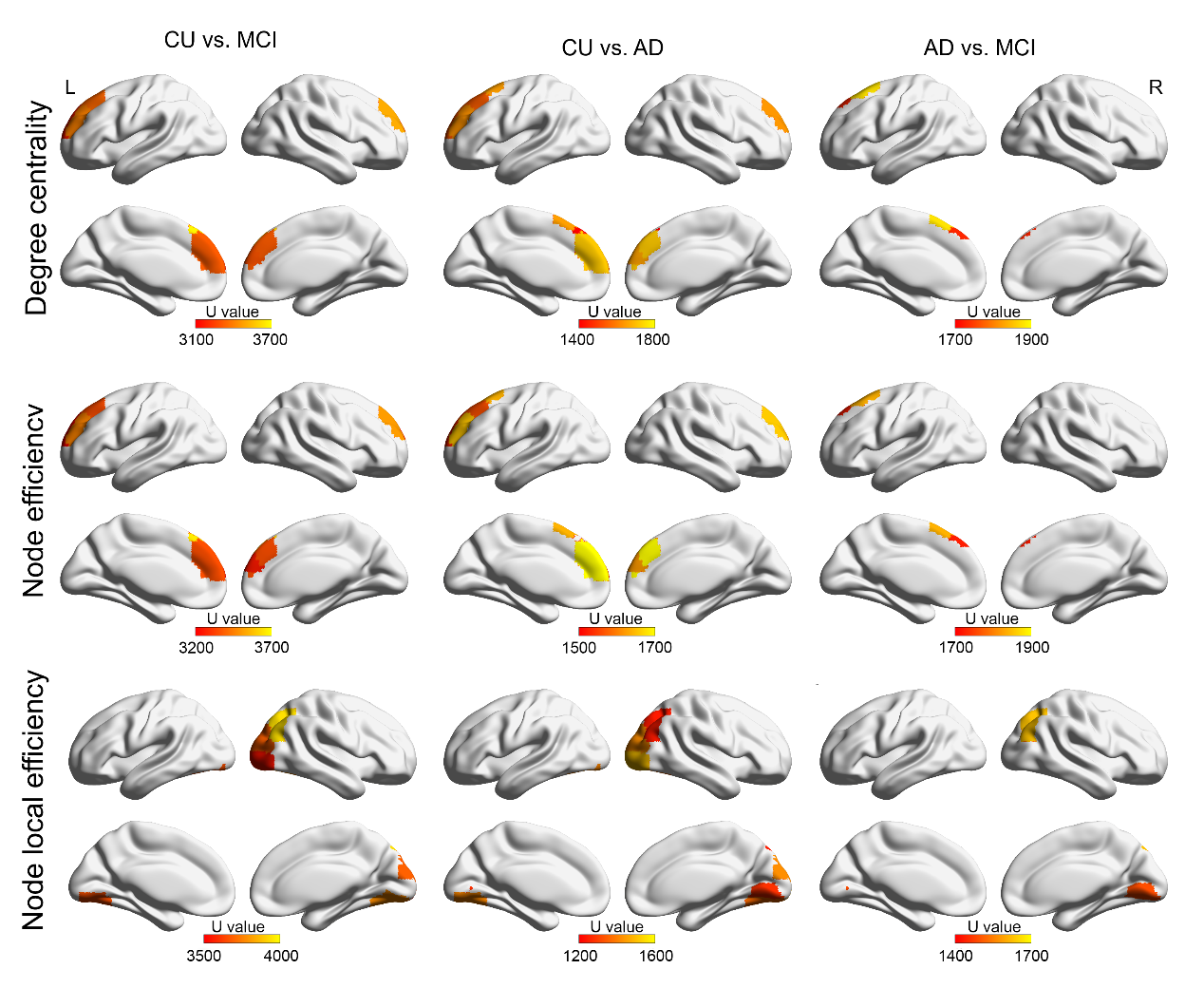


Figure S9. The statistical results of different graph theoretical parameters between CU, MCI and AD groups. The colorbar shows the statistical U values of Mann-Whitney U test.


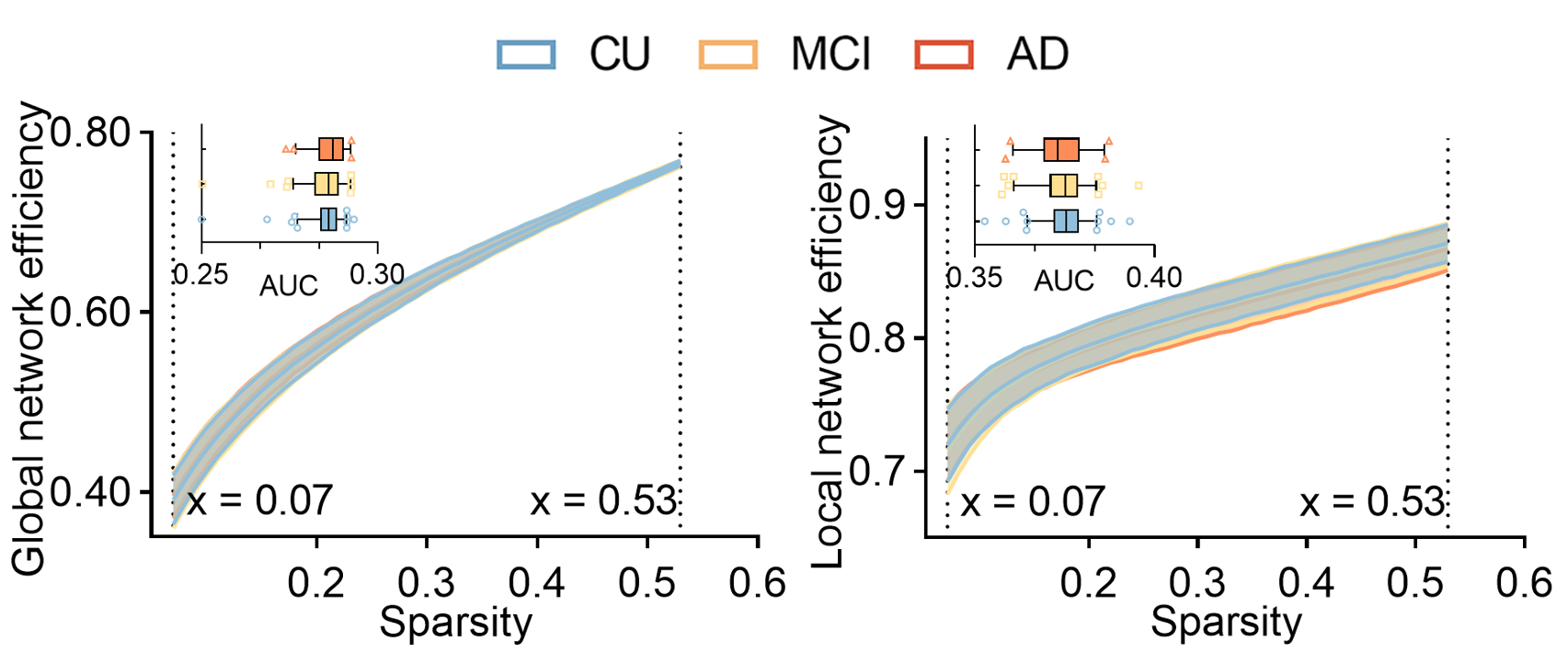


Figure S10. The result of network efficiency. The left and right sides showed the global and local network efficiency, respectively.

Table S2. The statistical information of dynamic measures in individuals with MCI and AD.

|  |  | MCI vs. CU | AD vs. CU |
| --- | --- | --- | --- |
| Persistence | State 3 | p = 0.005, U = 3430, \|d\| = 0.238 | p = 0.010, U = 1976, \|d\| = 0.254 |
| Resilience | State 3 | p = 0.005, U = 3437, \|d\| = 0.236 | p = 0.008, U = 1957, \|d\| = 0.262 |
| Transition probability | | | |
| State 2 → State 5 | | p = 0.023, U = 3648, \|d\| = 0.189 | p = 0.023, U = 1863, \|d\| = 0.297 |
| State 3 → State 3 | | p = 0.005, U = 3437, \|d\| = 0.236 | p = 0.008, U = 1957, \|d\| = 0.262 |

|d| represents the cliff delta.
